# Advancements in Transmitters and Sensors for Biological Tissue Imaging in Magnetic Induction Tomography

**DOI:** 10.3390/s120607126

**Published:** 2012-05-29

**Authors:** Zulkarnay Zakaria, Ruzairi Abdul Rahim, Muhammad Saiful Badri Mansor, Sazali Yaacob, Nor Muzakkir Nor Ayub, Siti Zarina Mohd. Muji, Mohd Hafiz Fazalul Rahiman, Syed Mustafa Kamal Syed Aman

**Affiliations:** 1 Biomedical Electronic Engineering Department, School of Mechatronic Engineering, University Malaysia of Perlis, Arau, Perlis 02600, Malaysia; E-Mails: s.yaacob@unimap.edu.my (S.Y.); hafiz@unimap.edu.my (M.H.F.R.); 2 Control & Instrumentation Engineering Department, Faculty of Electrical Engineering, Universiti Teknologi Malaysia, Skudai 81310, Johor Bahru, Malaysia; E-Mails: ruzairi@fke.utm.my (R.A.R.); saiful.home@yahoo.com (M.S.B.M.); normuzakkir@mail.com (N.M.N.A.); 3 Biomedical Engineering Unit, Engineering Services Division, Ministry of Health, Malaysia, Level 2-5, Blok E6, Parcel E, Precinct 1, Federal Government Administrative Centre, Putrajaya 62590, Malaysia; E-Mails: szarina@uthm.edu.my (S.Z.M.M.); smkamal@moh.gov.my (S.M.K.S.A.)

**Keywords:** tomography, biological tissue, magnetic induction tomography, transmitter, sensor

## Abstract

Magnetic Induction Tomography (MIT), which is also known as Electromagnetic Tomography (EMT) or Mutual Inductance Tomography, is among the imaging modalities of interest to many researchers around the world. This noninvasive modality applies an electromagnetic field and is sensitive to all three passive electromagnetic properties of a material that are conductivity, permittivity and permeability. MIT is categorized under the passive imaging family with an electrodeless technique through the use of excitation coils to induce an electromagnetic field in the material, which is then measured at the receiving side by sensors. The aim of this review is to discuss the challenges of the MIT technique and summarize the recent advancements in the transmitters and sensors, with a focus on applications in biological tissue imaging. It is hoped that this review will provide some valuable information on the MIT for those who have interest in this modality. The need of this knowledge may speed up the process of adopted of MIT as a medical imaging technology.

## 1. Introduction

Magnetic Induction Tomography (MIT) which is also known by the name of Mutual Inductance Tomography or Electromagnetic Tomography (EMT) is among the technologies ventured in the early 90s with first report appearing in 1992–1993 [1]. Like other modalities, the research has involved both the process industry [2–8] and biomedical tissue imaging, which this article is going to focus on. The MIT modality is categorized as a passive imaging modality together with Electrical Impedance Tomography (EIT) [9–13], Electrical Capacitance Tomography (ECT) [14,15] and Magnetostatic Permeability Tomography (MPT) [16–18]. All these modalities are sensitive to all three passive electromagnetic properties which are conductivity, permittivity and permeability of the material where in this article the interest is on biological tissues.

Several studies based on magnetic induction applications to biological tissues had been reported in 1968 by Tarjan and McFee [19] followed by Netz *et al.* [20] and Al-Zeibak and Saunders [21]. Their works have been continued by the new researchers who made MIT of interest to many researchers around the World with the new innovations and discoveries. Among the applications involved are lung monitoring and imaging [20,21], brain imaging and stroke related problem [20,22–28], liver tissue monitoring [29–31]physiological measurement [27] and several others not listed here.

Through contributions by Gabriel *et al.* [32] who had mapped out the range of suitable frequencies for biological tissues based on the experiments done by previous researchers, the interest in MIT research had gained some positive sides. One motivation for researchers who are involved in these passive electrical properties is their characteristic dependence on the state of hydration of biological tissue [23,25,29,31,32]. This provides an opportunity and alternative in studying the human body based on passive imaging modalities.

The aim of this review is to discuss the challenges of the MIT modality and summarize the recent advancements in transmitters and sensors, with a focus on applications in biological tissue imaging. It is hoped this review will provide some valuable information about the fundamental and current progress of MIT hardware (sensors, transmitters and electronic parts) for the researchers and those interested in this modality. The need of this knowledge may speed up the process of MIT of being among the adopted technologies in medical imaging.

## 2. MIT Theoretical Concepts

**Figure 1.**
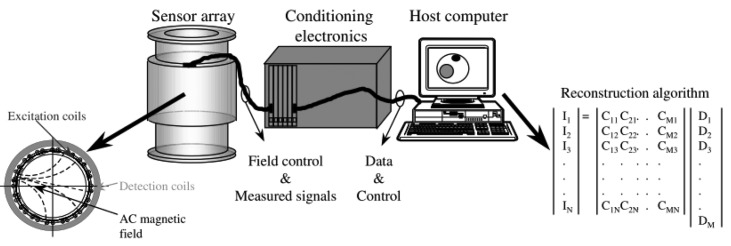
Block diagram of a typical MIT system illustrated by Binns *et al.* [3].

**Figure 2.**
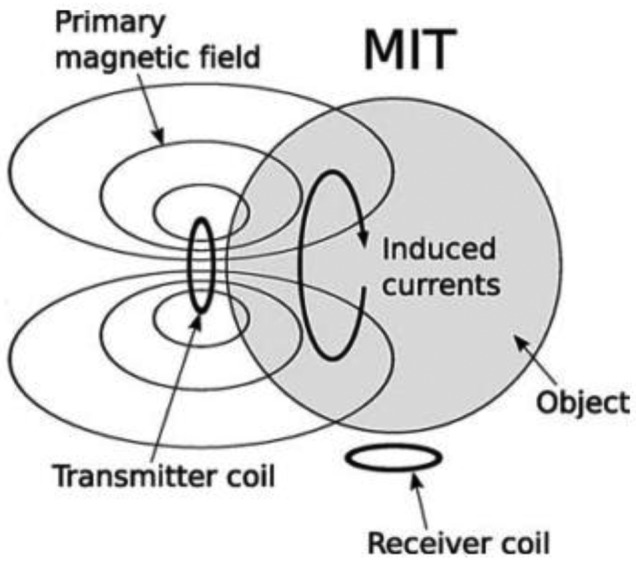
Principle of a MIT system illustrated by Gursoy *et al.* [45].

MIT is a low resolution imaging modality which aims at reconstruction of electrical conductivity, permittivity and permeability in the object [1,8,17,23,33], which is similar to the more established technique of Electrical Impedance Tomography (EIT) [9,10,34–36]. In biological tissues, the conductivity component is always dominant compared to permittivity and permeability [1,37–39] as the permittivity term for biological tissues is much smaller than the conductivity, especially at frequencies within the β-dispersion range (10 kHz–10 MHz) [40]. In term of devices used, MIT is different from EIT since it does not require galvanic coupling between the device and the object, hence avoiding the ill defined electrode-skin interface [25,29,37–39]. MIT instruments consist of several components which are sensors (excitation coils, detection coils, and screen), interface electronics and host computer [3] as shown in Figure 1. This contactless technique applies the interaction concept of an oscillating primary, **B_0_** generated by excitation coil with the conductive medium (object under investigation). This interaction is accompanied by the induction of eddy currents in the medium itself as the primary field propagates and penetrates the medium. The field due to these eddy currents is known as the secondary field, Δ**B** and also known with the name of magnetic perturbations field [1,19,39,41–44]. All these fields are sensed by the sensors at the receiver side. The concept of MIT is shown in Figure 2.

**Figure 3.**
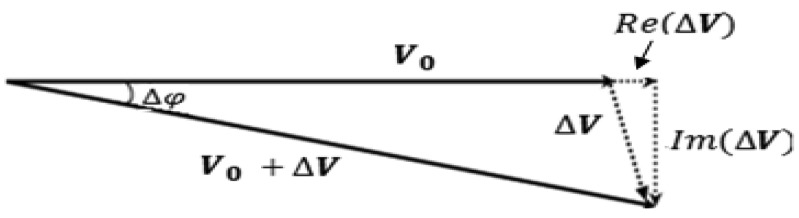
Phasor diagram of the MIT received signal [1].

In term of signal view, this can be explained through the phasor diagram shown in Figure 3. At the receiver, the total received signal is **V_0_** + **ΔV**, where **V_0_** is the signal induced direct from the primary field, **B_0_** at the primary coil, while **ΔV** is the signal derived from eddy currents field (secondary field, Δ**B**) induced within the investigated object and the phase angle is Δ*φ*. The skin depth, *δ* of electromagnetic field in the material (strictly for a plane wave) is given by:

(1) $$\delta ={\left(\frac{2}{{\omega \mu }_{0}\mu_{\text{r}}\sigma }\right)}^{\frac{1}{2}}$$

where ω is angular frequency; *μ*_0_ is permeability of free space; *μ_r_* is relative permeability of the sample and σ is the conductivity of the sample [39,46]. In biological tissues, skin depth is always large compared to the thickness of the sample, hence the secondary field is nearly 90^°^ phase shifted with respect to the primary field [1]. In relation to that, the **ΔV** signal which carries the information on the electrical properties of the material [where the *Re* (Δ***V***) and *Im* (Δ***V***) components represent permittivity and conductivity of the object, respectively is essential for the solution of the inverse problem and will be considered in the image reconstruction [42,43].

The carried information is on the changes of *k*, the complex conductivity distribution of the medium which is given by:

(2) k=σ+jωɛ

Changes *Δk* will change the value of **ΔB**, hence this change will automatically affect the value of **ΔV** [49]. For a biological tissue equivalent sample (assuming μ_r_ = 1, σ≫ ωε) the secondary signal **ΔV** will be proportional to the frequency and sample conductivity [25,27,43].

## 3. Challenges in MIT

A great challenge in an MIT system for imaging biological tissue is that the primary field **B_0_** is much larger than the secondary field, with a Δ**B** of the order of 10^2^–10^6^ times greater, depending on the frequency of operation and coil geometry [35]. This phenomenon is due to the relatively low conductivity of the tissue [35,45]. Griffiths [46] had noticed that the expected perturbation of the received signal due to conduction of eddy currents within biological tissues, which is 1% of the primary signal, was still small, even with the use of high frequency (HF) excitation fields (10 MHz). Through single channel measurement, Watson *et al.* [48] had reported that the practical benchmark for biomedical MIT was to be able to resolve 1% variations in the field perturbations in the biological tissues whereas for tissues with conductivities in the biological range of 0.1 S/m–2 S/m, the maximum phase shift expected was of the order of 1° at 10 MHz.

Moreover, the primary field existing at the receiver has introduced noise into the signal measurements [47–50]. The noise can be in two forms that were by restricting the gain which may be applied to the received signal and thereby increasing the contribution of quantization errors, and secondly by introducing phase noise and drift errors in the in-quadrature signal [51]. The errors may be obvious with the existence of unwanted electric-field (capacitive) coupling between the excitation coils and the sensors. Even though this coupling does not contribute to noise, it may cause a systematic error that remains constant in the MIT measurements [52]. These phenomena become worse for low conductivity materials such as biological tissues [39,51,52]. On the other hand, noise may also appear from the thermal motion of free electrons in the measuring apparatus [7,46,53–55]. Due to that, corrective action needs to be considered during experiments for minimizing or eliminating these sources of errors.

## 4. Techniques to Overcome

Several steps and techniques have been taken by researchers to overcome this challenge in minimizing these major issues on the receiver signals. Among the methods that have been introduced were gradiometer (axial & planar), electromagnetic screen (shielding), magnetic-confinement screen, coil orientation, enhancement in electronic circuit and also through the use of multi-frequency techniques.

### 4.1. Gradiometer

A gradiometer is an instrument that is used in measuring the gradient (numerical rate of change) of a physical quantity, such as a magnetic field. It is used in the elimination of influence from ambient fields which exist in the measurement. In the MIT case, through the use of a gradiometer, most of the primary signal is cancelled in the sensor itself. The advantage of gradiometer is that the sensor can remove most of the primary signals effects which contain phase noise, thus it gives a high primary signal that affects the cancellation factor [1], besides being mechanically very stable. There are three types of gradiometers; axial, planar and asymmetrical. However only axial and planar gradiometers are discussed in this article since the asymmetrical gradiometer is not so popular in the MIT application [56].

#### 4.1.1. Axial Gradiometer

**Figure 4.**
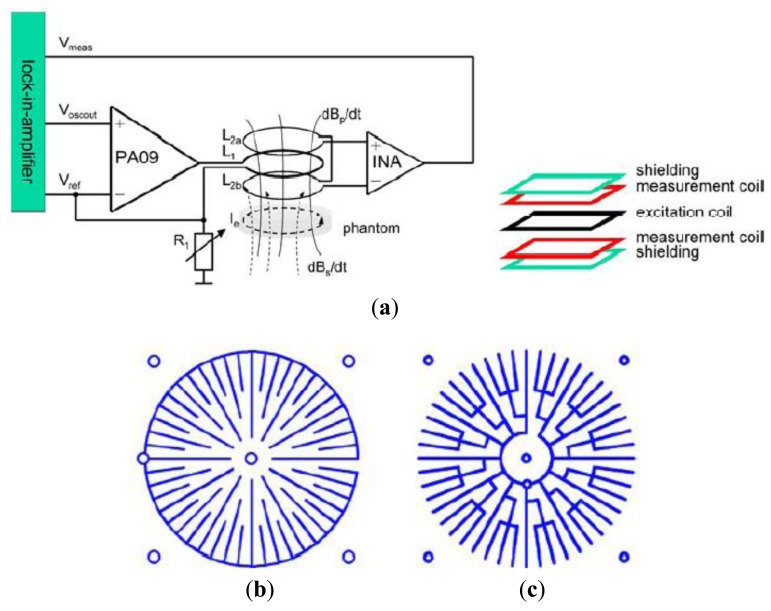
An axial gradiometer developed by Riedel *et al.* [59]. (**a**) The measurement system with arrangement of shielding; (**b**) Shielding with circle type; (**c**) Shielding with star type.

Tarjan and McFee [19], Netz *et al.* [20] and Scharfetter *et al.* [57] had implemented this symmetry method in cancelling the primary field. Through this technique two coils were located at equal distances at any axis of symmetry with the primary coil at the middle. Karbeyaz and Gencer [58] in their research had implemented a single coaxial gradiometer which can moved over the phantom using an XY scanning system. In a more proper design, Riedel *et al.* [59] had studied the precision and sensitivity of an axial gradiometer consisting of five PCBs. In avoiding capacitive coupling, these PCBs were covered with shielding layers on top and bottom, followed by measurement coils, with the excitation coil located in the middle as in Figure 4(a). In this study, two types of shielding; circular type and star type (see Figure 4(b,c)) were compared through measurement. The purpose of radial shielding was to avoid circular eddy currents from disturbing the measurement. The study had shown a linear variation in both types of shielding with the sensitivity of 0.003 mV·S^−1^·m at 600 Hz. However noise and drift were in the same range at lower current value (154 mA) for imaginary and real in both shielding types. 2 × 2 planar matrix arrangements proved that distance between adjacent sensors provided significant sensitivity, however major improvements were still required in noise and drift.

Xu *et al.* [60] had implemented in their multichannel hemispherical glass bowl measurement systems what Riedel and his group had done before, but with several modifications to the circuit. They had employed an independent single cancellation sensor in improving the system's stability and decreased the phase drift caused by the ambient temperature variation and other influences. They had also included difference amplifier with high CMRR for capacitive coupling rejection, whereas shielded cables had been proposed in avoiding some unwanted signals to couple into the signal channels.

#### 4.1.2. Planar Gradiometer

Ketchen *et al.* [61] had demonstrated the first thin-film planar gradiometers consisting of parallel and series configured pickup loops directly coupled to the superconductivity quantum interference device (SQUID) inductance. The development of efficient planar coupling schemes for dc SQUIDs in 1981 led to the development of improved thin-film planar gradiometers with an intrinsic balance of better than 1 part in 10,000 and a projected factor of 100 improvement of the magnetic field gradient noise sensitivity. Poor intrinsic balance causing them to remain significantly sensitive to the fields they had been design to reject.

With several enhancements Stolz *et al.* [62] introduced a planar gradiometer design with long 4-cm baseline consisting of two series-configured pickup loops transformers coupled to a thin-film SQUID with magnetic field gradient noise ranged from 0.36 to 0.72 fT/cm-Hz½ and intrinsic balance in the range from 10^−4^ to 10^−3^.

**Figure 5.**
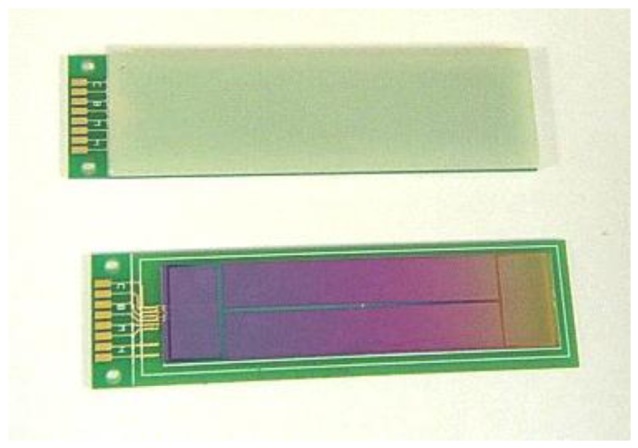
Thin film planar gradiometer developed by Cantor *et al.* [63].

Cantor *et al.* [63] had developed a thin-film planar dc SQUID gradiometer shown in Figure 5 where the design was the same as developed by Stolz, but with a featured contact pad placement at the ends of the chip rather than in the middle. The gradiometer featured smooth dc characteristics, low noise performance and can be operated without shielding in typical laboratory environments without losing lock. This type of gradiometer was attractive for biomedical imaging and other applications requiring low-noise measurements in noisy environments.

**Figure 6.**
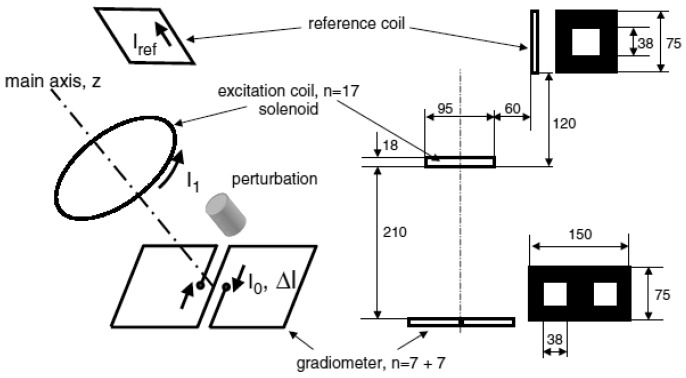
COIL-PGRAD system developed by Scharfetter *et al.* [27].

Scharfetter *et al.* [27] had introduced an adjusted planar gradiometer (PGRAD) with planar geometry (See Figure 6). The design exhibited an anti-symmetric sensitivity with respect to left-right transverse axis (axial symmetry), and thus was sensitive to the location of perturbation in the z-axis whereas it was insensitive to the object in the x-y plane.

This single channel measurement in combination with a high resolution phase detector had compared the performance of PGRAD with coil as a sensor in term of signal to noise ratio (SNR) and signal to carrier ratio (SCR). The receiver coil was arranged in the same position as the excitation, where primary signal was subtracted from the captured signal using the value measured by reference coil. SNR is given by $\frac{\Delta V}{e_{n}}$ whereas SCR is $\frac{\Delta V}{V_{0}}$ [40], *e_n_* is voltage noise in the amplifier. They had reported that planar gradiometer has an improved SNR value of 34.1 dB compared to only 12.9 dB if a coil were used, which was 164.3% better. This showed that through the fabrication of gradiometers in PGRAD on the same PCB board, their intrinsic balance was improved since the process was estimated to be accurate to a certain degree of accuracy. This high intrinsic balance had given PGRAD more capability in reducing the noise compared to coils through minimizing the primary field. This was also reflected in the signal to carrier ratio (SCR) which has increased about 20 dB if using PGRAD due to the significant reduction of **V_0_**. Quite low SNR and SCR of receiver coil may be related to the imperfect subtraction of the primary field at the receiver coil itself due to the manually adjusted back-off coil in the absence of an object.

Using the same setup as in Figure 6, Rosell *et al.* [40] had evaluated analytically and experimentally the performance of a planar gradiometer as a sensing element in an MIT system with both sensors (receivers) fabricated on PCB. They had identified the major advantage of a planar gradiometer compared to a coil was the bigger measured phase shift produced by the perturbation while the major problem was that it was insensitive to objects with axial symmetry due to its anti symmetrical response. In comparison to coils, planar gradiometers provided a robust and stable cancellation technique, capable of reducing the carrier signal in the absence of conductivity perturbations while maintaining essentially the same absolute sensitivity for local perturbations. This produced a bigger relative sensitivity of the gradiometer when compared to a coil. Results showed that a system using a planar gradiometer as detector has less demanding requirements for the electronic system than a system using simple coils. In addition, a preliminary study of the sensitivity matrix for an imaging system with 16 gradiometers showed a decrease in the matrix rank compared to a system with 16 coils.

Still with the same single measurement setup as in Figure 6 but only using PGRAD as receiver, Scharfetter *et al.* [31] had done experiments which aimed at two objectives; brain edema monitoring and estimation of hepatic iron stores in certain pathologies. Four important error sources had been studied that were moving conductors near the PGRAD, thermal drifts of the PGRAD-parameters, lateral displacements of the PGRAD and phase drift in the receiver. They had noticed that all errors affected the detected real part (mainly related to ε and μ) of the measured complex field was more than the imaginary part (mainly related to σ), which was up to 62 times an SCR for aluminum sphere moving case. Thermal mismatches of 0.15 K at 150 kHz between both gradiometer halves introduced $\text{Im}(\frac{\Delta \text{V}}{{\text{V}}_{0}})$ of 10^−7^ whereas for $\text{Re}(\frac{\Delta \text{V}}{{\text{V}}_{0}})$ the value was 50 times larger. In term of small lateral displacements of receiver, for 10 μm displacement can caused $\text{Re}(\frac{\Delta \text{V}}{{\text{V}}_{0}})$ by a factor of more than 1000 compare to $\text{Im}(\frac{\Delta \text{V}}{{\text{V}}_{0}})$ which was not more than 10^−7^.

**Figure 7.**
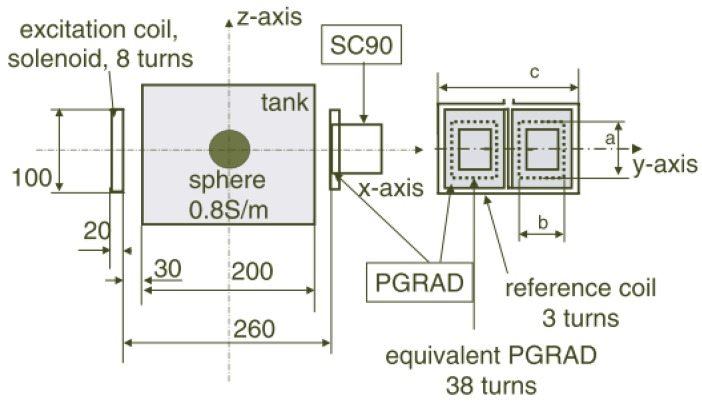
New single channel MIT system developed by Scharfetter *et al.* [64] for comparing the performance of PGRAD and solenoid coil as receiver.

Again, in comparing the effectiveness of PGRAD and solenoid coil as a receiver, Scharfetter *et al.* [64] developed another single channel MIT system shown in Figure 7. In difference to the earlier setup in Figure 6, the receiver coil was arranged with its axis perpendicular to the excitation coil in such a way that the receiver was insensitive to the primary signal, thus the measured signal at the receiver was only due to the presence of an object. They had reported the advantages of PGRAD which much less sensitive to far sources of electromagnetic interference thus suitable for an open system without screen. The drawback was it had problems with phase errors due to thermal mismatches between two gradiometers halves. However for coils, besides its simpler design which can be fabricated and calibrated easily, the advantages came when the system was shielded against RF sources, since coils had worse immunity to electromagnetic interferences.

**Figure 8.**
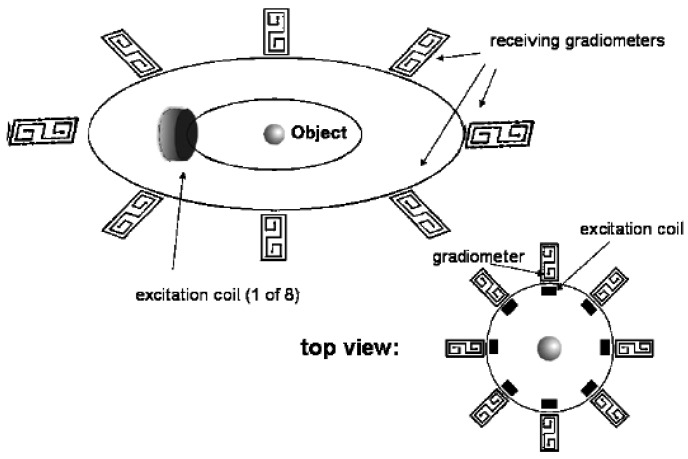
ZFGRAD system developed by Scharfetter *et al.* [65].

In a more advanced version of the previous design, Scharfetter *et al.* [65] had developed a multi channel MIT imaging system with the introduction of new gradiometer known as zero flow gradiometer (ZFGRAD), seen in Figure 8. ZFGRAD which combined the advantages of PGRAD and zero flow coil (ZFC) where ZFC was also known as Bx sensor [51]. PGRAD had the capability of cancelling the interferences from far RF to a high degree through differential design of both halves, whereas ZFC can easily be positioned in a perpendicular orientation with respect to the excitation coil in such a manner that it has zero net primary magnetic flow in it. ZFGRAD was proven to have better immunity to far magnetic perturbations compared to PGRAD and ZFC (relatively up to 2 and 12 times better). In terms of sensitivity it was the worst among the three, however the morphology of the sensitivity maps for the three types were very similar, where ZFC and ZFGRAD exhibit their maximum sensitivities near the tank borders on the side of the excitation coil whereas the PGRAD was more sensitive near the receiver side. The slight differences in the sensitivity maps are only due to the different geometries. In addition, for a multi channel MIT, it was hard to adjust all gradiometers to be nulled to the primary signals for all excitation coils, thus there were still residual signals that exist which in turn contributed to noise in the measurements. Like receiver coils, whereby even small errors in the perpendicular positioned angle of the coils provided opportunity for primary signals to penetrate the coil hence contributed additional value to the true measured secondary signals. In this case, normalization for measurement at every excitation cycle is vital in reducing the errors due to this imperfect position of the sensors.

**Figure 9.**
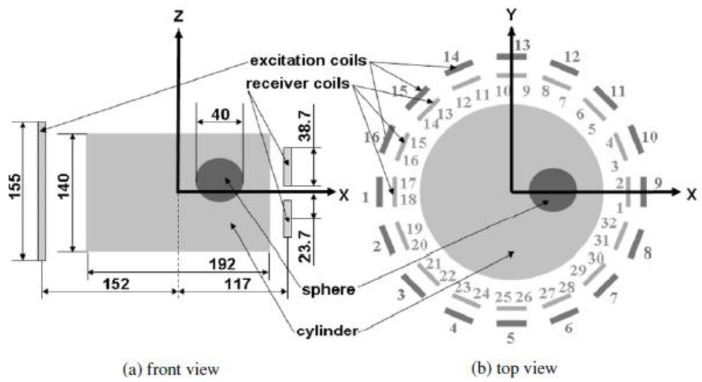
System modeled by Merwa *et al.* [66].

In different view, Merwa *et al.* [66] had simulated a model consisting of 16 excitation coils and 32 receiver coils which could be combined pairwise to give 16 planar gradiometers as illustrated in Figure 9. The simulation compared the performance of 32 receiver coils with 16 planar gradiometers. Reconstructed images using 32 receiver coils showed a good localization of the perturbed sphere but all perturbations are slightly displaced towards the nearest border of the cylinder. In contrast, 16 planar gradiometers (combination of two parallel coils) produced a better localization; however there were two places in which ghost images with opposite sign mirrored the true image with respect to the x-y plane.

**Figure 10.**
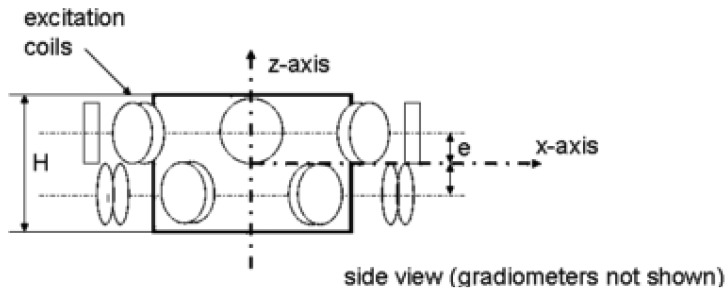
Two rings system proposed by Scharfetter *et al.* [65] to overcome the ‘ghost image’ issue which is due to symmetry of the receiver coils.

This was due to symmetry of the coil itself which is unable to distinguish between a positive conductivity on the upper half region and the negative conductivity on the lower half if the same corresponding conductivity detected, since the induced ***Δv*** in both coils was always the same. This effect can be eliminated by placing two parallel rings of planar gradiometers around the cylinder which was either one ring should be rotated to some angle relative the other one, in order to achieve a lower degree of symmetry [65] as in Figure 10. On the software side, Merwa and Scharfetter [34,35] proved that Point Spread Function (PSF) had the capability of solving this ‘location unrecognized’ issue, since PSF which defined the propagation in an image due to a point source or point object depend on the location and geometry of the perturbation.

### 4.2. Electromagnetic Screen

The application of electromagnetic screens in the MIT hardware system may overcome the existing capacitive coupling between excitation coils and receiver coils [67] which generates noise in the measured signals, while a magnetic confinement screen helped in improving the sensitivity of the system. There are several types of electromagnetic screens: outer screen, inner screen and electro-magnetic capsule [7,39,48,68–72].

**Figure 11.**
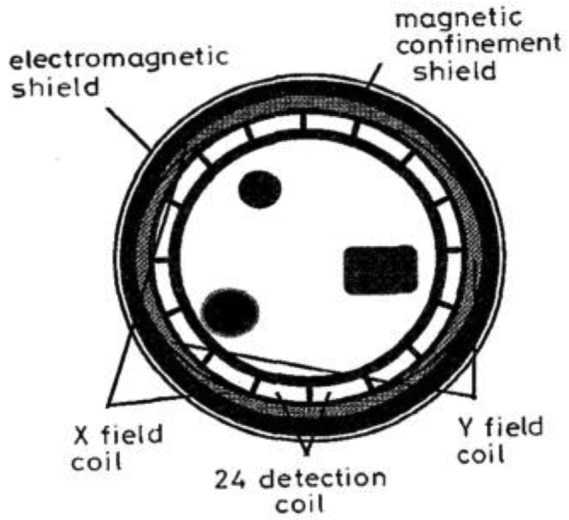
Two types of screen used by Peyton *et al.* [68].

Peyton *et al.* [68] and Yu *et al.* [73] had introduced two types of screen which they called outer electrical screen and magnetic confinement shield in their system (see Figure 11). The magnetic confinement shield constructed from a ferrite powder/polypropylene composite material, concentrated the field inside the image space and prevented interference from external sources. They also had located the electronic circuit in a capsule to provide more shielding to the circuit.

Igney *et al.* [71] did include in their experiment on a planar-array MIT system, an electric field shield constructed from a copper strip board placed on top of each excitation coil. The electric-field shielding employed therefore reduced the electric-field coupling by a factor of approximately 4. The results suggested that significant electric-field coupling still remained, even with the application of electric field shielding.

**Figure 12.**
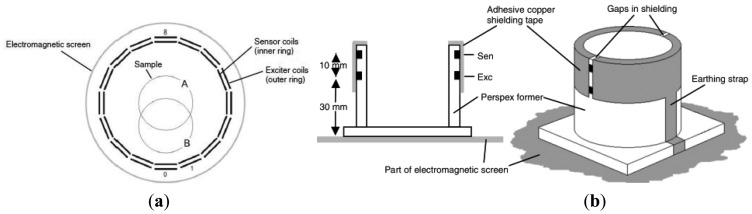
Electromagnetic screen implemented by Griffifths *et al.* [39]. (**a**) Plan view; (**b**) Cross section view and pictorial view.

Griffifths *et al.* [39] had enclosed their MIT system within an aluminum electromagnetic screen with the coils themselves screened individually (see Figure 12). The coils were mounted on Perspex formers inside the electromagnetic screen with two gaps left in the screen to prevent eddy currents circulating in it. The electromagnetic screen was proven to reduce the relative permittivity of water from 87 ± 6 (without screen) to 81 ± 6 (with screen) which indicated the effectiveness of the outer electromagnetic screen in eliminating capacitive coupling by ‘attracting’ the electric field.

**Figure 13.**
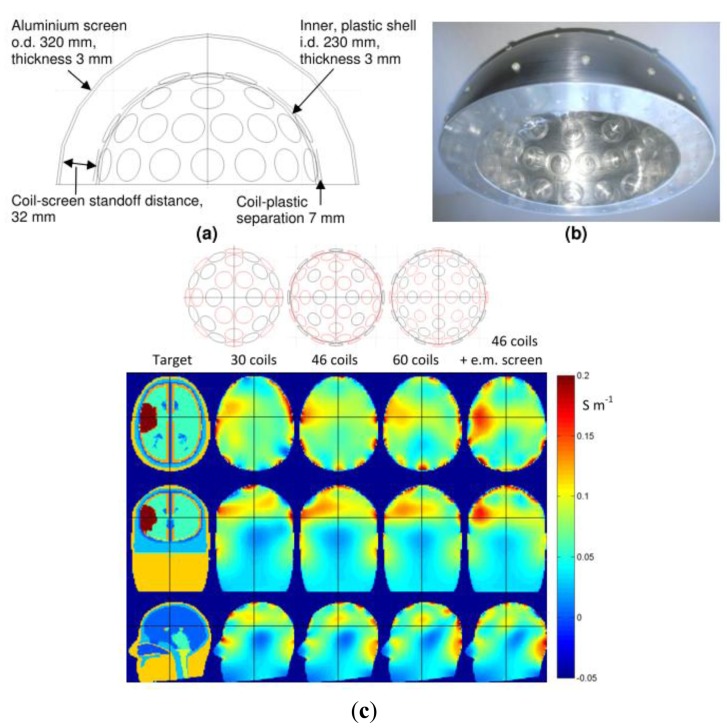
Semi hemisphere screen of Cardiff MK2b head array implemented by Griffifths *et al.* [70]. (**a**) Final dimension of helmet; (**b**) Helmet prior to adding the electronics; (**c**) reconstructed images based on simulated data on different number of coils and with electromagnetic screen.

In a different design for the purpose of cerebral haemorrhage imaging, Griffifths *et al.* [70] had implemented an aluminum hemisphere screen in their frequency-difference helmet array system (see Figure 13) where the helmet inner size conformed closely the shape of a normal adult's head. The standoff distance for the screen was of 60–80% of the coil diameter which provided reasonable suppression of inter-coil capacitive coupling without excessively damping the inductive signals. Compared to a conventional cylindrical array, this hemispherical design which consisted of arrays could increase the inductive coupling to the brain and hence the sensitivity to conductivity changes within it. By locating the coils as close as possible to the scalp, the MIT signals would be maximized. This was more obvious with the increasing of the number of excitation coils however the drawback was also the increase of the capacitive coupling effects. From simulation they proved that the screen was effective in rejecting interference and minimizing inter-coil capacitive coupling where the results showed through the implementation of electromagnetic screen on 46 coils, the visualization of the stroke region was more visible compared to the unscreened system, even with increasing receiver numbers.

**Figure 14.**
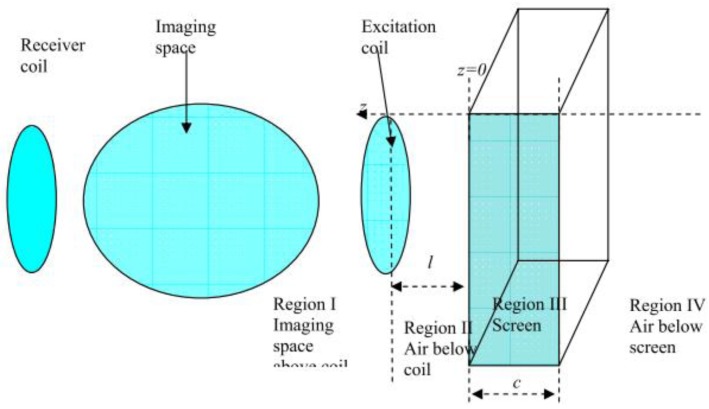
Electromagnetic screen model developed by Yin *et al.* [69].

Yin *et al.* [69] did mentioned that in high frequency and low conductivity applications, the design of the outer screen needs to take into account the design parameters of the screen (thickness, distance to coil, materials) in improving the performance of the sensor system. From their analysis based on Figure 14, they had reported that in avoiding significant reduction, it had to be at least three times the diameter away from the coil. The thickness of the screen also played an important role as if it was much less than the skin depth, the screen still has a significant effect on the sensitivity maps. At low frequency, thick screens allowed EM fields to penetrate the material due to skin depth effects, which in turn produced eddy current fields which opposed the primary field, thus reducing the net EM field near the screen. A very thin screen (thickness less than skin depth) would not let the EM field penetrate into the screen, hence it induced an eddy current field at the surface of the screen which acted as a barrier to the internal and also the external field. This effect would be more pronounced when a higher frequency was used.

### 4.3. Excitation Coil Design

**Figure 15.**
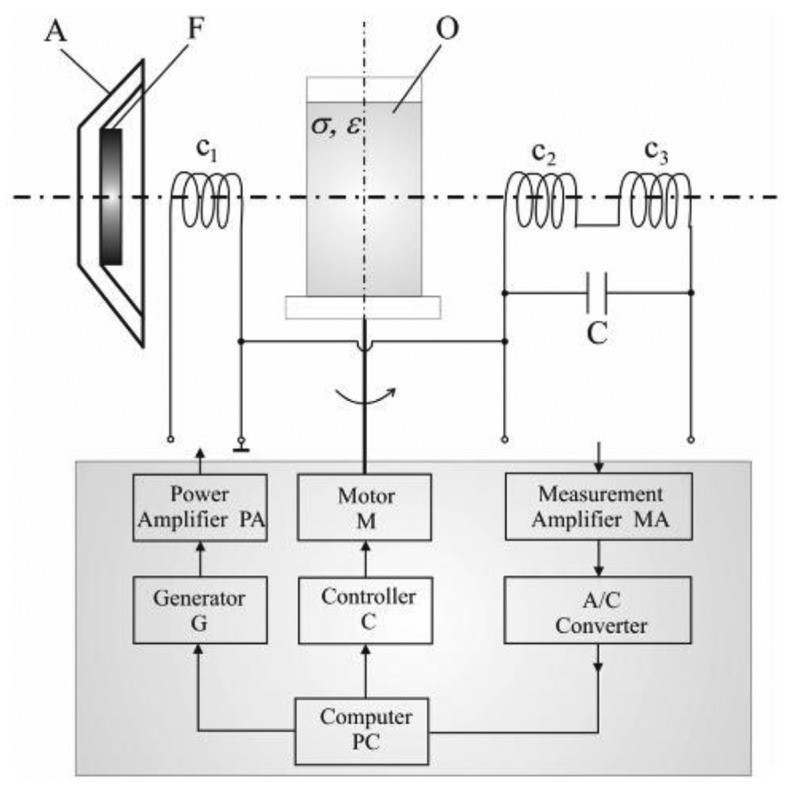
Coil system produced by Stawicki *et al.* [74] where A is the excitation coil screen, F is ferrite core and O is the object.

Other than the abovementioned techniques, excitation coil design also plays an important role in minimizing the effect of the primary field [72,73]. Stawicki *et al.* [74] had proposed an exciting coil design as seen in Figure 15, which has a conducting shield to protect the primary field from scatter around and also to the outside. A ferrite core which has high permeability relative to the surrounding air, is located at the centre of the screen and is capable of concentrating the primary magnetic field lines in the core material itself. The presence of the ferrite core which made this design different from others, could increase the magnetic field of a coil by a factor of several thousand compared to without the use of the core.

**Figure 16.**
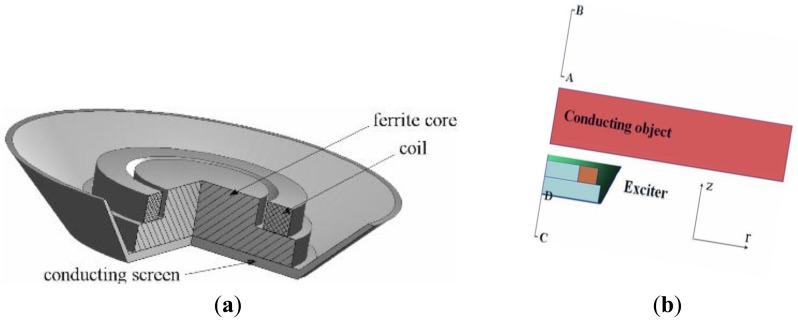
(**a**) Exciting Coil system produced by Stawicki *et al.* [74]; (**b**) Device model by Barba *et al.* [75].

Barba *et al.* [75] had almost the same design as Stawicki and his group. They had explained the objective of their design through the diagram in Figure 16(a). Based on the diagram, the axial induction field B_z_ should be maximized (along line A–B) while it should be minimized along line (C–D) as in Figure 16(b). Related to that, more proactive action had been taken in their experiment where each electronic modules for excitation and receiving coil were mounted at the outer wall of the tank and each was placed in a separate metallic case for shielding purposes [76].

### 4.4. Sensor Arrangement

**Figure 17.**
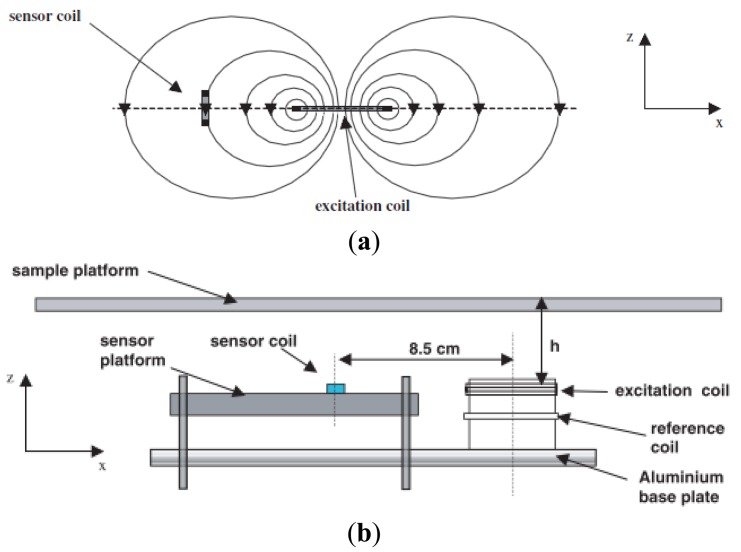
Sensor-coil alignment system by Watson *et al.* [77]. (**a**) Sensor-coil alignment for zero sensitivity concepts; (**b**) measurement platform.

In MIT, the location of the receivers affects the quality of the image reconstruction in terms of primary field effect cancellation. Watson *et al.* [77] had suggested that primary field compensation for a planar array can be done through a sensor coil (Bx sensor) which is aligned in such a way that it provides zero sensitivity to the excitation field because no magnetic flux threads it. The system as in Figure 17 employed a 10 μH surface mount chip inductor with a ferrite-cored miniature solenoid and provided suitable sensitivity and resonant characteristics over the frequency range 1–10 MHz. Through this sensor, the noise and drift in the signal were reduced by factors of 43 and 51 respectively, relative to the uncompensated orientation. The large improvement in noise and drift performance was due entirely to the reduction in the sensitivity of the sensor to the primary excitation field.

**Figure 18.**
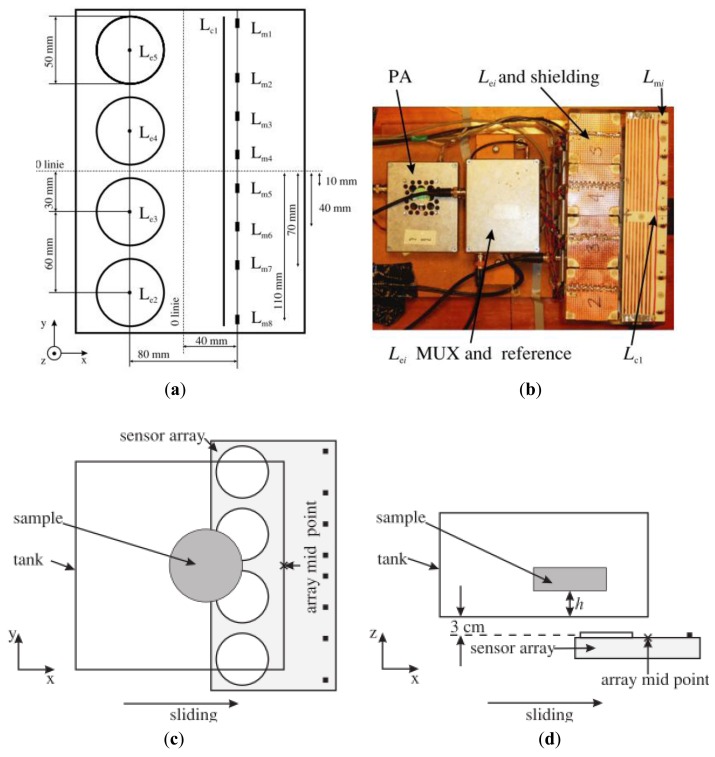
Planar-array system developed by Igney *et al.* [71]. (**a**) Sensor positioning and numbering; (**b**) Picture of the developed system; (**c**) Plan view; and (**d**) side view.

Igney *et al.* [71] enhanced the experiment done by Watson *et al.* [77] by changing the normal excitation coil to a shielded PCB printed excitation coil as shown in Figure 18. They had reported that the planar array was found to provide flux-linkage minimization of the primary field for all channel combinations, on average by a factor of around 20, and ranging from 50 for channel combinations in which the excitation and measurement coils were close to each other. Larger improvements in noise and drift, by factors of 14 and 27, respectively, were observed in the real component. The system was found to provide a SNR of 30–50 dB over the frequency range 1–8 MHz, based on the peak value for all channels. This higher SNR was contributed to by the accurately designed PCB printed excitation coil and the use of a surface mount inductor as sensor, compared to hand winding with which it was very difficult to get sufficient accuracy. Accurate design provided the excitation coil-Bx sensor with perfect alignment, hence improving the efficiency of the insensitivity to the primary field effects.

**Figure 19.**
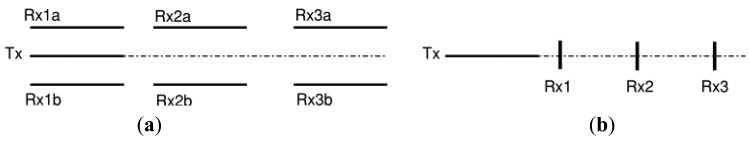

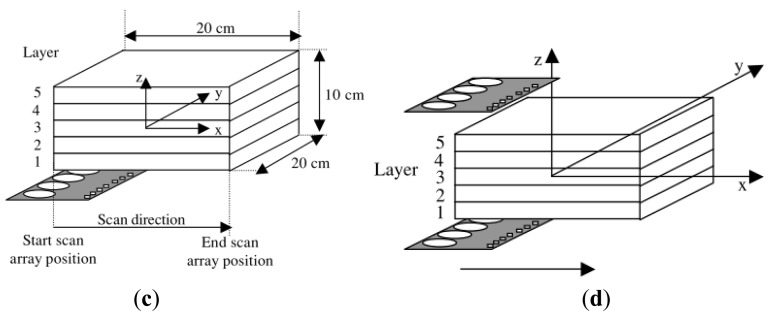
Experimental illustration of Watson *et al.* [51] on planar-array system. (**a**) Gradiometer; (**b**) Bx sensor; (**c**) Array scan for single plane geometry; and (**d**) Array scan for two-plane geometry. Tx was excitation coil.

Watson *et al.* [51] had examined the relative performance of axial gradiometers and coil-orientation methods (Bx sensors) through computer simulation of the sensitivity profiles produced by a single sensor and comparison of reconstructed images produced by sensor arrays. The developed system was almost the same as developed by Igney *et al.* [71] but with some additional features as two plane-arrays had been included (see Figure 19). They had suggested that the Bx sensor provided better sensitivity at depth compared to the axial gradiometer and may be the most suitable sensor for measurements of electrical impedance within one excitation coil radius into the sample. However if surface measurements were required with the depth sensitivity limited to the surface layer, e.g., measurement of electrical impedance of epithelium, then axial gradiometer appeared to be more suitable.

**Figure 20.**
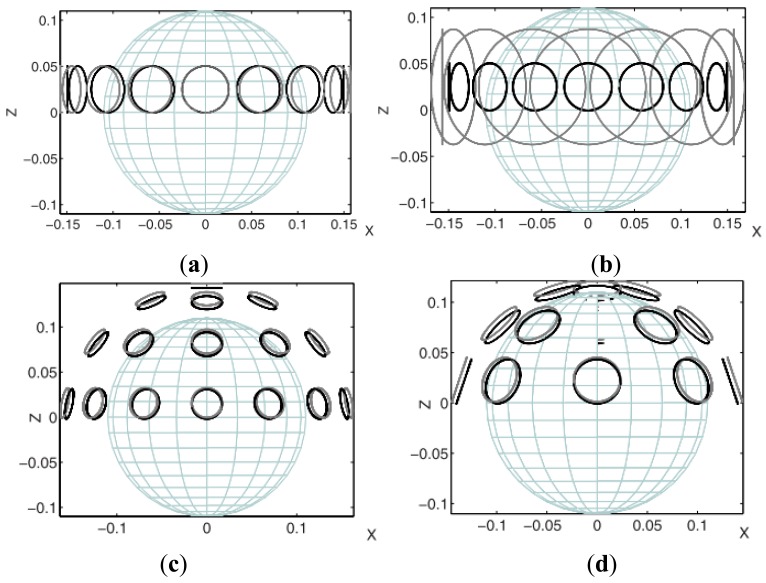
Experimental setup developed by Eichardt *et al.* [78]. (**a**) Model A1; (**b**) Model A2; (**c**) Model B1; and (**d**) Model B3. The setup B2 uses 16 pairs of coils arranged corresponding to B3, but with coil sizes equal to B1.

Eichardt *et al.* [78] evaluated and compared through simulation the cylindrical and the hemi-spherical coil setups of two Magnetic Induction Tomography (MIT) systems using sensitivity analysis. Two models of cylindrical and three models of hemi-spherical (see Figure 20) have been simulated that identified the influence of the number and area parameters of the coils on the sensitivity to conductivity changes. Their findings indicated that the hemi-spherical MIT system with a smaller distance between the layer of coils and the measurement object showed a clearly higher sensitivity compared to the cylindrical MIT system. In addition, the two simulated setups with larger coil areas provided higher sensitivities in relation to the standard setups, while the difference between the hemi-spherical setups using a different number of coils with identical areas was relatively small. They had also reported that generally, there was a considerably strong decay of the sensitivity values for an increasing distance between the elements and the coils. Their study showed differences of up to seven orders of magnitude within the upper hemispherical volume of interest (VOI) considering a specific setup. In term of hemi-spherical design, the simulated system (Figure 20(c,d)) was quite similar to that of Griffiths *et al.* shown in Figure 13, however there was no application of aluminum screen where both excitation coils and receiving coils were located, on the other hand the excitation coil simulated by Eichardt did position it just above the receiving coil.

**Figure 21.**
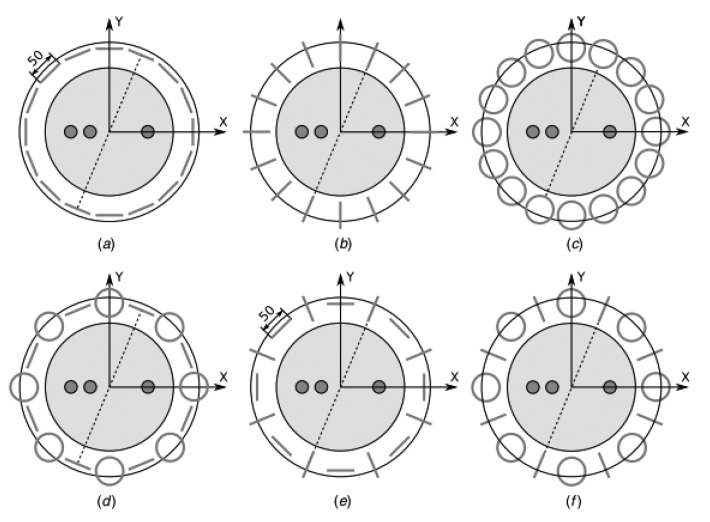
Coil orientations tested by Gursoy and Scharfetter [79]. Six types of corresponding design (**a**) D1; (**b**) D2; (**c**) D3; (**d**) D4; (**e**) D5; and (**f**) D6.

Gursoy and Scharfetter [79] had studied a number of receiver array designs with different suggested coil orientations and singular value decomposition (SVD) was used as a basis for the analysis. Six different designs (D1–D6, see Figure 21) had been demonstrated and evaluated at five SNR different values while at each SNR value, each design was obtained as a truncation level. The images corresponding to the investigated designs were reconstructed by using the noise-free and noisy data to present the artifacts in the images. It was found that the proper choice of the coil orientations significantly influenced the number of usable singular vectors and the stability of image reconstruction, although the effect of increased stability on the quality of the reconstructed images was not of paramount importance. It was found that, each design has its own merits and shortcomings for different imaging regions and for different SNR levels. However in considering overall characteristics, D1, D2 and D5 were found to be more focused to the median plane with high resolution and low image uncertainty. For the off-median regions, D4 was found to be moderately better among the others considering the practical noise levels of MIT, from 20 to 40 dB.

In another study, Gursoy and Scharfetter [80] had introduced a fast deterministic algorithm to obtain optimum receiver array designs for a given specific excitation. The design strategy developed was based on the calculation of the sensitivity matrix. Therefore, no voltage data simulation or noise considerations were needed to obtain optimal designs. Through this algorithm, iterative exclusion of receiver locations that yield poor conductivity information from the space spanning all possible locations was done, until a feasible design is reached. MIT designs that are currently used in the existing hardware were evaluated, and it was shown that better designs can be achieved for different excitation and receiver patterns. They had stated that the method did not guarantee finding the global optimum, however, a close approximation is possible by a good initial discretization of the receiver geometry. The algorithm was also capable of finding a design that focuses onto a region inside the body in increasing the image resolution at that region.

**Figure 22.**
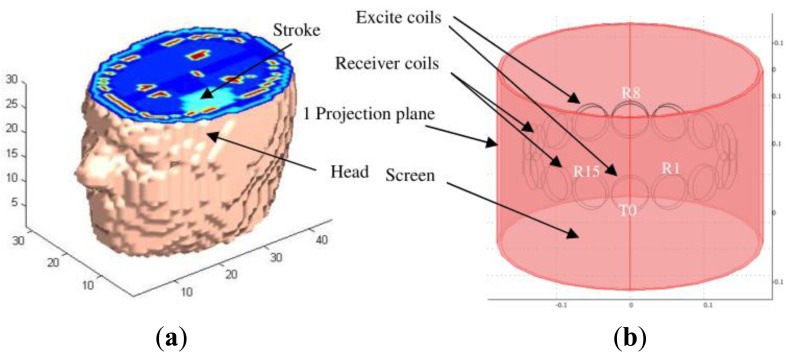
Simulation model developed by Dekdouk *et al.* [81]. (**a**) Phantom of head model and (**b**) Model of the MIT system.

Dekdouk *et al.* [81] had done a simulation based on a model of a head as phantom and an MIT system to investigate the capability of frequency difference in reducing the error caused by coil positioning. The design included of a cylindrical shield and a circular coil array which consisted of 16 excitation coils and 16 receiver coils. Both excitation and receiver coils were modeled as filamentary and arranged in two concentric circles at different radius surrounding the target (see Figure 22). The applied frequencies were 1 MHz and 10 MHz. The results had shown that there were no improvements on the results of the errors due to coil positioning relative to single frequency measurement; hence this inferred that there was no advantage to using frequency difference for sensor displacement error cancelation.

**Figure 23.**
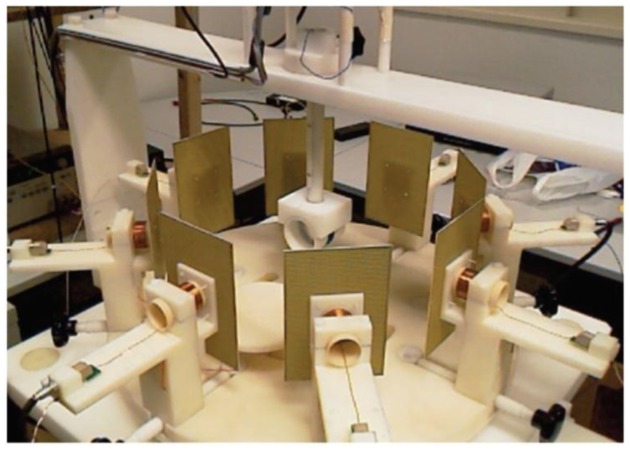
New system developed by Bras *et al.* [82].

Bras *et al.* [82] had came out with their new MIT system as in Figure 23 with recent improvements in the measured signal stability and accuracy as well as a much improved angular resolution measurement of the multi-coil setup. This prototype had been packaged together with a new mechanical design consisted of single excitation coil and eight moving sensing coils which functioned to rotate and to move the body vertically. Each pair of opposite sensing coils was directly connected at a pre-amplification circuit input. The coils could be mechanically positioned in order to obtain the least residual signal possible for each sensing coil pair. The mechanical system was made in a PVC material, electrically and magnetically inert and there were no metal structures in an approximately 1 m diameter around the source coil.

The used source AC current was 500 mA operated at 870 kHz. This system allowed obtaining longer stable and more accurate acquisitions, improving the number of measurements without trends or external perturbations which leads to a better conductivity resolution and to an enhanced image reconstruction. It had several advantages over the classical circular setup: (i) the sensor position error due to movement was not as critical as in the case of a standard setup; (ii) the carrier amplitude was not varied considerably along positions, meaning that due to its symmetry no position was preferential. Finally, (iii) it allowed for differential measurements for better excitation field effects suppression, as was the case of planar gradiometers.

**Figure 24.**
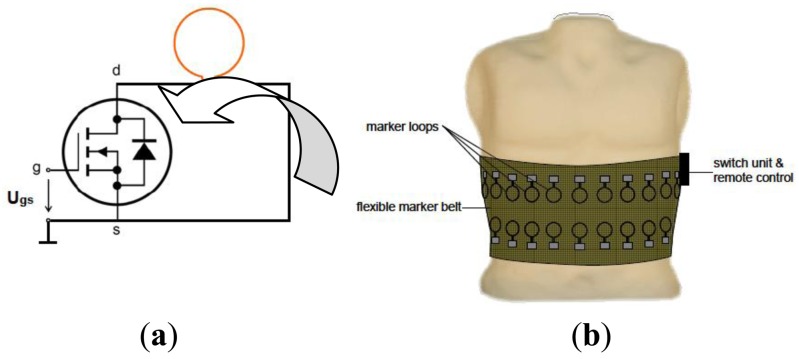
Active marker system developed by Scharfetter *et al.* [83]. (**a**) Active marker design; (**b**) Schematic of an active marker system in form of an elastic belt with several loop/switch units which were controlled remotely from the data acquisition control unit.

Scharfetter *et al.* [83] had developed a new technique as in Figure 24 for artifact suppressions during object movements through the use of object movements tracking signal which was directly from the MIT system through the application of active markers.

The basic idea was to place the active markers (which consist of small loops of a very thin wire which can be opened and shorted via a tiny switch) on the surface of the body with the positions were chosen such that the markers were in front and very close to gradiometers which located in the zone of maximum sensitivity of the coils. The achieved simulated images showed that a reasonably accurate reconstruction of the markers can be achieved when assuming an SNR which was closed to that determined experimentally. This proved that tracking of object boundaries by only using the MIT signal was feasible. However, further investigations need to be done to find the most appropriate marker designs and measurement frequencies in achieving the optimum results.

### 4.5. Types of Sensors Used

Types of sensor used in the measurement will also determine the accuracy of the results since magnetic field measurement can be done through the use of several types of sensors. The most common and famous one was coils, but there were several researchers who proposed Hall effect element component [84] while some were interested in SQUID (Superconductivity Quantum Interference Device) for non-destructive evaluation [85,86], besides magnetic sensors [87]. In most sensors, the practical limit of the resolution depended on the possibility of achieving the noise floor. The smaller the noise floor level, the resolution was much better [56]. Noise floor was the measure of the signal created from the sum of all the noise sources and unwanted signals within a measurement system, where noise is defined as any signal other than the one being monitored.

#### 4.5.1. Coil

**Figure 25.**
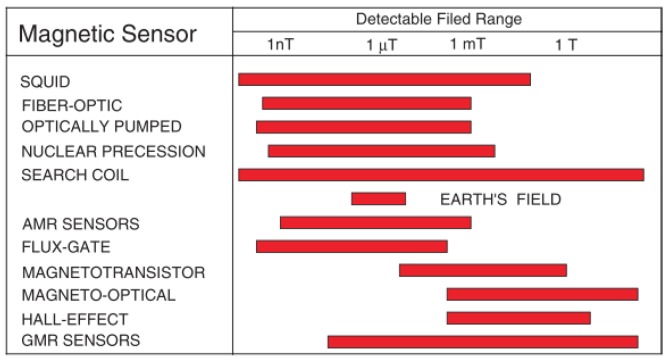
Typical field range of various magnetic field sensors [56].

Coils had been used by most of the researchers in MIT whereby the magnetic field measurement component was based on electromagnetic induction theory. Coils are sensitive only to the flux that was perpendicular to their main axis. Tumanski [56] did mention that induction coils which are also known as search coils, pickup coils or magnetic antenna were one of the oldest and best known types of magnetic sensors. In term of detectable field range, he had concluded that coils were the best compared to others (see Figure 25).

#### 4.5.2. Hall Effects Element

Hall effects elements as the magnetic field measurement component are based on the Hall effect concept. The measurement range of Hall effect elements was mainly from 10 gauss to several thousand gauss, making them an ideal choice to measure large magnetic fielda but not precise enough to detect eddy current induction fields. The operation frequency range of Hall effect elements was from 20 kHz to 100 kHz, so they are not suitable to detect high frequency magnetic fields [88]. The noise floor level of Hall sensors was ∼10 nT Hz^−1/2^ [56].

#### 4.5.3. Superconductivity Quantum Interference Device (SQUID)

**Figure 26.**
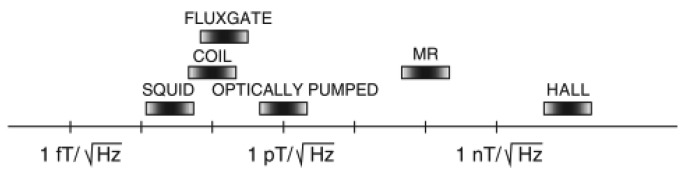
Typical resolutions of various field sensors [56].

A SQUID is an extremely sensitive magnetic flux-to-voltage transducer [85] consisting of two superconductors separated by thin insulating layers to form two parallel Josephson junctions. The device may be configured as a magnetometer to detect incredibly small magnetic fields at less than pT level (See Figure 26). The noise floor level of SQUID was ∼50 fT Hz^−1/2^ [83]. Krause *et al.* [89] had developed a pulsed eddy current NDE technique through the application of a High Temperature Superconductor (HTS) SQUID which allowed simultaneous analysis of the sample at all depths. The advantage of SQUID magnetometers over induction coil sensors was that the field did not decay as rapidly as its time derivative, allowing for a broader range of investigated depths. In a coil, the field derivative dB/dt decayed much faster and therefore, reached the noise level much earlier than the transient of the field itself. This means, compared to a coil, a magnetometer sensor potentially can record the transient data at later times. Anyhow compared to other sensors the SQUID was quite expensive [88].

#### 4.5.4. Magneto-Resistive Sensors

Magnetic sensors make used of the magneto-resistive effect whereby a magnetic material changes its resistance in the presence of an external magnetic field. There was no dB/dt dependence unlike in coils. They are high precision, have a wide frequency range and quite low cost. Liu *et al.* [88] had proposed a Honeywell HMC1021Z magnetic sensor in his MIT research. This sensor has a Set/Reset function which can eliminate the magnetic field disturbances outside. The noise floor level of magneto-resistive sensors is ∼100 fT Hz^−1/2^ compared to that of coils which is less than 100 fT Hz^−1/2^ [56].

### 4.6. Electronic Circuit

**Figure 27.**
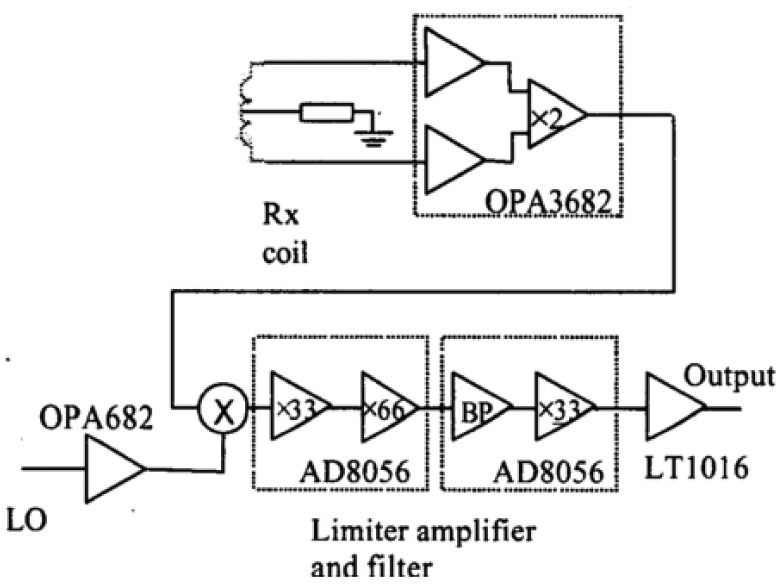
Receiver circuit developed by Watson *et al.* [48].

Korjenevsky *et al.* [42] had reduced the electric coupling through common mode rejection by the differential inputs at the receivers. However no detailed circuits and results on the electric coupling reduction had been reported. Watson *et al.* [48] had used the same common mode rejection technique as Korjenevsky, but providing some explanation of the circuit diagram of the receiver circuit as in Figure 27. They had used OPA3682, an instrumentation amplifier with a gain of two as receiver front end, allowing conversion of the received signal from balanced to unbalanced while providing some rejection of capacitive coupling. This capability was due to the disable functions of the OPA3682 which placed the inputs into a high impedance state, allowing isolation of the transmitter coil when not in use, thus cutting off the current from flowing in the excitation coil and so to avoid capacitive coupling. The output signal was then mixed with local oscillator signal and then further went to three stages of amplification of 97 dB in total. The filtering process used a first order band pass filter of −3 dB attenuation at 8 kHz and 12 kHz. However no detailed analysis on noise elimination performance was reported.

In term of phase drift measurements, Watson *et al.* [54] had found that the vector-voltmeter system showed a performance advantage by a factor of 2 and this was likely due to the superior stability provided by digital filtering compared to analog filtering. It was also reported that a phase error existed during the time delay between measuring the frequency of the reference and demodulating the received signal with the synthesized reference, that was greatly reduced when the uncompensated crystal oscillators in the transmitters were replaced with temperature compensated ones. Watson *et al.* [77] in their new study had reported the small surface mount inductors were found to produce satisfactory performance in terms of noise, drift and linearity and were readily available and inexpensive.

**Figure 28.**
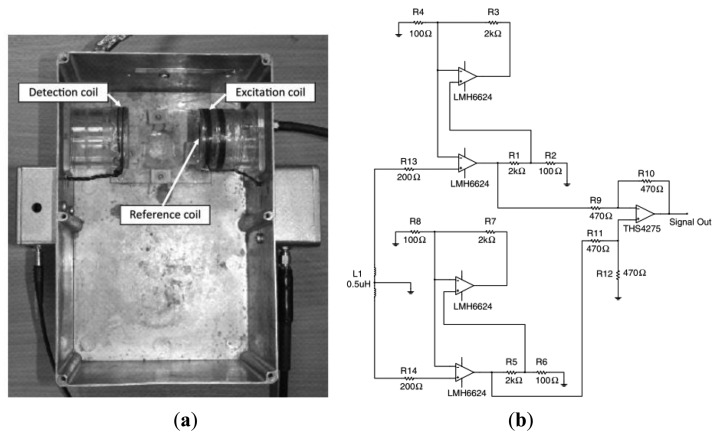
MIT spectrometer system developed by Watson *et al.* [29]. (**a**) Hardware of the experiment; (**b**) the detector circuit.

Latest, Watson *et al.* [29] had introduced a highly phase stable differential detector amplifier for magnetic induction tomography for the purpose of achieving the required phase measurement precision. To reach this objective, he and his team had developed an ultra-phase-stable, low noise instrumentation amplifier with proven average change of −0.1 ± 0.6 m·°C^−1^ as the ambient temperature was varied between 35 and 50 °C, with gain of 21 at 10 MHz operational frequency as in Figure 28.

### 4.7. Multi-Frequency Technique

The multi-frequency technique in MIT is the application of multiple frequencies during operation. This technique is capable of reducing the acquisition time and drift errors [7], while producing better sensitivity in different regions within the object [72]. This is because errors in the absolute values do not affected the results strongly, due to separating system drifts and temporal changes of the conductivity data [31].

Scharfetter *et al.* [31] had developed a magnetic induction spectroscopy (MIS) system as in Figure 7 where the explanation on the system performance also had been discussed. They had suggested that the exploitation of multi-frequency information should be done through the implementation of Cole-Cole parameters in separating the system drift and temporal changes of conductivity data, which has more to do with signal processing than hardware as is the focus of this article.

**Figure 29.**
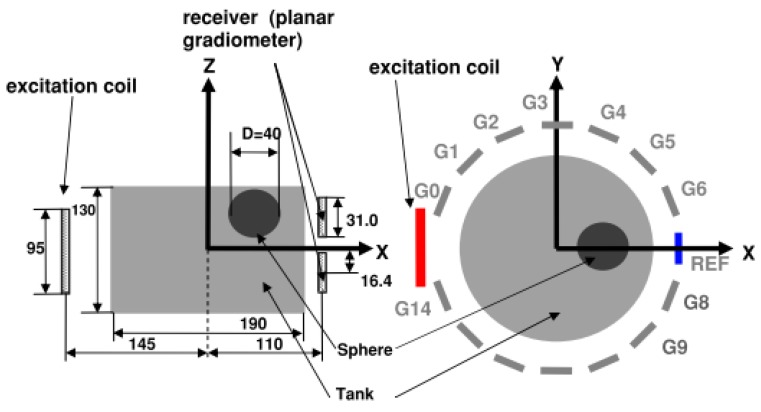
Multi-frequency system design developed by Ferrer *et al.* [7].

Ferrer *et al.* [7] had developed 14-channel multi-frequency (50 kHz–1 MHz) magnetic induction tomography system (MF-MIT) where the excitation field was produced by a single coil and 14 planar gradiometers were used for signal detection (see Figure 29). The real and imaginary parts of ***ΔB/B_0_*** were calculated using coherent demodulation at all injected frequencies. They had found that for long acquisition times the drift in the signal produced a bigger effect than the input noise (typical STD was 10 nV with a maximum of 35 nV at one channel), but this effect was reduced using a drift cancellation technique based on averaging.

Brunner *et al.* [41] had implemented a differential multi-frequency technique in their system with the objective of reconstructing the shape of the conductivity spectra. Two reference frequencies had been used, 100 kHz and 300 kHz. Through this differential character method, the spectra did not provide absolute conductivities but preserved the shape of the spectrum. In term of artifacts, the occurrence depended on the sensitivity matrix and the regularization parameter chosen, but it always exists. In term of errors, the use of different reference frequencies should minimize the errors because of the better validity of the ‘small perturbation’ assumption with increasing frequency, for example at lower conductivity steps.

In conclusion, multi-frequency is self-referencing and allows changes in conductivity with frequency, particularly of biological tissues, to be measured directly, without the need for another reference. In addition, errors in the assumed geometry tend to cancel as they will be the same for all frequencies.

## 5. Future Research Aspects

Based on the review, it can be seen that MIT system is an interesting research area that needs to be explored. There are still many ways MIT systems can be improved, even with the aggressive research that has been carried out by previous and current researchers with successful outcomes.

Further development on the applied sensors, jig design and also electronic circuits are needed since these are the front end of the system which is crucial in data collection. The study on material and shape of screen of excitations coil together with suitable specification and geometry for coil parameters may help in boosting optimum focusing capability of the primary field on the object of interest, hence increasing the value of the secondary field generated by the object itself. The design of the sensor jig also needs to incorporate the real scale of the dimension in such a way that it could be used for clinical purposes in future imaging instruments. The future research also should take into account the anisotropic properties of biological tissues, since this issue has to be addressed in real clinical imaging instruments.

Image reconstruction algorithms also cannot be put behind, since very good quality images not only depend on the measurements and signal processing side, but also on great design on the image reconstruction algorithm side. The algorithms should not only be limited to linear types, but should also focus on the non-linear or semi-linear which may produce useful clinical results either in 2D, 3D or both, for static and/or dynamic imaging of the body.

On the other hand, the design also has to consider the real world problem of the errors due to body movements and unspecified physiological changes, for example the body temperature and sweating.

## 6. Conclusions

In this article, challenges and recent advances on sensor and transmitters for the MIT technique are described. Several techniques have been introduced in solving or eliminating the primary field effects and noise problems that occur at the receiver side which is the great challenge in biological tissue imaging due to its very low conductivity. The action taken covers both sides; transmitters and also receivers. On the transmitter or excitation side, screens have been introduced to the excitation coil where this may focus the primary field on the region of interest while minimizing the scattered field to the neighboring circuits. On the receiver side, gradiometers, Bx sensors, sensor arrangement techniques, selection of high sensitivity sensors and also the introduction of highly stable phase detector differential amplifiers have had a positive outcome in improving the performance of the MIT systems. However further research need to be carried out in enhancing and upgrading the current MIT systems to make them more fascinating and applicable as a real imaging system in the medical imaging industry.
